# Development of a peptide targeting dopamine transporter to improve ADHD-like deficits

**DOI:** 10.1186/s13041-018-0409-0

**Published:** 2018-11-09

**Authors:** Terence K. Y. Lai, Ping Su, Hailong Zhang, Fang Liu

**Affiliations:** 1 Campbell Family Mental Health Research Institute, Centre for Addiction and Mental Health, Toronto, ON M5T 1R8 Canada; 2 Department of Physiology, University of Toronto, Toronto, ON Canada; 3 Department of Psychiatry, University of Toronto, Toronto, ON Canada; 4 Institute of Medical Science, University of Toronto, Toronto, ON Canada

## Abstract

Attention-deficit hyperactivity disorder (ADHD) is a neurocognitive disorder characterized by hyperactivity, inattention, working memory deficits and impulsivity. Its worldwide prevalence is estimated to be 3–5% in children and adolescents. The mainstay treatment for ADHD is stimulant medications (e.g. methylphenidate), which increase synaptic dopamine by directly blocking dopamine transporter (DAT). Although these pharmacological agents are effective, they are often associated with various side effects including risks for future substance use disorders in ADHD patients. Here, we investigated an interaction between DAT and dopamine D2 receptor (D2R) as a novel target to develop potential therapeutics for the treatment of ADHD by using an interfering peptide (TAT-DAT_NT_) to dissociate this protein complex. We found that TAT-DAT_NT_ promotes locomotor behavior in Sprague-Dawley rats. Furthermore, using in vivo microdialysis and high-performance liquid chromatography, we found that the disruption of D2R-DAT elevates extracellular dopamine level. More importantly, the interfering peptide, TAT-DAT_NT_, attenuates hyperactivity and improves spontaneous alternation behavior in spontaneously hypertensive rats (SHR) ------ a common animal model of ADHD. This work presents a different means (i.e. other than direct blockade by a DAT inhibitor) to regulate the activity of DAT and dopaminergic neurotransmission, and a potential target site for future development of ADHD treatments.

**Electronic supplementary material:**

The online version of this article (10.1186/s13041-018-0409-0) contains supplementary material, which is available to authorized users.

## Introduction

Attention-deficit hyperactivity disorder (ADHD), characterized by hyperactivity and inattention, affects between 3 and 5% in children and adolescents worldwide [1, 2]. Although the exact etiology of ADHD remains elusive, dysregulation of the dopaminergic system is heavily implicated due to the actions of the current ADHD treatments [3]. These pharmacological agents are stimulants such as methylphenidate [4], and they enhance dopaminergic neurotransmission by directly blocking dopamine transporters (DAT) [5]. Although they are effective clinically, there are concerns about potential drug abuse and risks for future substance use disorders [6–8]. Radiolabelling studies revealed that both methylphenidate and cocaine share similar binding patterns within the dopaminergic system (e.g. nucleus accumbens, etc.), which is implicated in euphoria and repeated substance use [9–11]. More importantly, chronic administration of these direct blockers leads to up-regulation of DAT expression [12, 13], which possibly underlie the increased risk for subsequent substance use disorders [14, 15]. Therefore, an improved ADHD treatment may strengthen dopaminergic neurotransmission without directly blocking DAT.

Dopamine is the predominant catecholamine in mammalian brain and is involved in neurological functions such as locomotion, cognition, emotion and reward [15, 16]. One of the key players in regulating dopamine signaling is the dopamine transporter (DAT). DAT belongs to the SLC6 family of Na^+^/Cl^−^ dependent transporters, and is composed of 12 transmembrane domains and intracellular N- and C-termini. With its localization on the presynaptic membrane of dopaminergic nerve terminals [17, 18], DAT utilizes the Na^+^ gradient created by the plasma membrane Na^+^/K^+^ ATPase as the driving force to transport synaptic dopamine across cellular membrane [19, 20]. Such reuptake action of DAT also serves to terminate dopamine signaling. The reuptake activity of DAT is greatly dependent on its membrane expression level, which is constantly under dynamic regulation. Furthermore, DAT can also be regulated through direct protein-protein interactions with intracellular proteins such as α-synuclein [21], synaptogyrin-3 [22], etc.

We previously identified a direct protein-protein interaction between DAT and dopamine D2 receptor (D2R) [23], and this interaction is mediated through the first 15 amino acids (i.e. M_1_-V_15_) of DAT N-terminus and the third intracellular loop of D2R. Functionally, through this interaction, D2R recruits DAT to plasma membrane and therefore enhances the reuptake of dopamine. Based on the amino acid sequence (M_1_-V_15_) of the interacting region in DAT, we developed a cell-permeable peptide (TAT-DAT_NT_) to disrupt this interaction.

Here we showed that the disruption of D2R-DAT interaction by TAT-DAT_NT_ peptide stimulates locomotor behavior in normal and dopamine-depleted Sprague-Dawley rats by increasing extracellular dopamine, suggesting the TAT-DAT_NT_ peptide might also exert beneficial effects in the animal model of ADHD. In agreement with our hypothesis, the administration of TAT-DAT_NT_ also rescues the hyperactivity, and improves spontaneous alternation behavior of spontaneously hypertensive rats (SHR) in a Y-maze, a commonly used animal model of ADHD. In summary, this study provides evidence to support the D2R-DAT interaction as a potential novel drug target for ADHD treatments.

## Methods

### Experimental animals

Three strains of male rats were included in this study: Sprague-Dawley (SD) rats, Wistar Kyoto (WKY) rats, and Spontaneously Hypertensive (SHR) rats. 8-week-old SD rats, 3-week-old WKY rats and 3-week-old SHR rats were respectively purchased from Charles River Laboratories (Montreal, Quebec, Canada). SHR rats originated at the Kyoto School of Medicine in 1963, where Okamoto mated an outbred Wistar Kyoto male with marked elevation of blood pressure to a female with slightly elevated blood pressure [24]. Young SHR rats are preferably used as an ADHD animal model, because they will become hypertensive at the older age. Upon arrival, with free access to food and water, animals were housed in a vivarium maintained at 22–24 °C room temperatures and on a 12:12 light-dark cycle. They were also given one week to acclimatize to the vivarium. All behavioral procedures were approved by the Animal Care Committee at Centre for Addiction and Mental Health.

### Drug

TAT-DAT_NT_ (YGRKKRRQRRRMSKSKCSVGLMSSVV) was commercially obtained from GenScript USA Inc. (Piscataway, NJ, USA), whereas TAT (YGRKKRRQRRR) control peptide was synthesized by Biomatik (Cambridge, Ontario, Canada).

### Surgery

Guide cannulae for intracranial injections (HRS Scientific, Canada) were accurately positioned to reach the designated coordinates (AP -1.0 mm, LM + 1.4 mm, DV –3.6 mm from bregma), and then secured in place with dental cement. Animals subjected to in vivo microdialysis had another guide cannula (SciPro Scientific Products & Equipment, Ontario, Canada) inserted into the following coordinates (AP + 2.2 mm, LM + 1.2 mm, DV –5.6 mm from bregma) to measure extracellular dopamine level in the core region of nucleus accumbens.

### Peptide-induced locomotor activity in SD, WKY and SHR rats

Animals were placed in open-field chambers for 30 min daily on two to three consecutive days, constituting their baseline locomotor activities. On the experiment day, animals received their peptide treatments (intracerebroventricular injection*; i.c.v*.). The i.c.v. administration was chosen to avoid potential bioavailability limitations. Thirty minutes after the peptide administration, animals were placed in open field chambers, and their locomotor activities were recorded for 30 min to an hour. For SD rats, animals received 40 nmol of TAT or TAT-DAT_NT_. For WKY and SHR rats, they were given a wash-out period of 3 days before they received a different dose (i.e. 0.4 nmol and 4.0 nmol) of peptide treatments. All peptide treatments were delivered intracranially.

### Acute dopamine depletion model in SD rats

This protocol was adapted and modified from McDougall et al. [25]. Animals from the AMPT group received two intra-peritoneal injections of AMPT (25 mg/kg each), separated by 2 hours. Subsequently, animals were placed in open-field boxes to track their locomotor activities for 30 min to confirm the validity of the model.

To examine the effects of TAT-DAT_NT_, animals were placed  in open field boxes for 15 min after the induction of dopamine depletion. Animals were then given an intracranial injection of 40 nmol TAT or TAT-DAT_NT_, and were immediately returned to open-field chambers for a 60-min recording session.

### Y-maze test

The Y-maze consisted of three arms made of black plastic (56 cm long, 12 cm wide and 25 cm high) extending from a central platform at an angle of 120°. Animals were administered with either TAT or TAT-DAT_NT_ (at the dose of 0.4 nmol; *i.c.v.*) 30 min before the Y-maze test. Subsequently, animals were placed at the end of one arm and allowed to explore freely among the three arms of the Y-maze for 8 minutes. An “arm entry” was made when an animal crossed one-third of the arm length from the central platform. The sequence of arm entries was observed and recorded. A successful alternation was defined as three consecutive entries into three different arms (i.e. A, B and C arms) such as ABC, ACB, BAC, BCA, CAB, or CBA. The percentage of spontaneous alternation behavior was determined as following:

$$ SAB\%=\frac{\#\mathrm{alternation}\times 100\%}{\#\mathrm{total}\ \mathrm{arm}\ \mathrm{entries}-2} $$

### In vivo microdialysis

Animals were anesthetized with inhalant isoflurane, and a microdialysis probe (MAB 9.14.2; SciPro, Canada) was inserted into the nucleus accumbens core. Throughout the experiment, animals were maintained under anaesthesia, and artificial cerebrospinal fluid (NaH_2_PO_4_ 2.0 mM, MgCl_2_ 1.0 mM, CaCl_2_ 1.2 mM, KCl 2.7 mM and NaCl 145 mM) was constantly pumped through the microdialysis probe at a rate of 0.5 μL/min. The dialysate samples were treated with 40 mM perchloric acid (PCA) and 5 mM EDTA before subjected to the high-performance liquid chromatography (HPLC). The baseline of each animal was determined based on four dialysate samples. After establishing the basal level of extracellular dopamine, peptide treatments were injected (*i.c.v*.) through the guide cannula, and five more dialysate samples were collected after the administration to evaluate the peptide effect on extracellular dopamine level. At the end of the experiment, brains were collected for cresyl violet staining to confirm cannula placement.

### High performance liquid chromatography (HPLC)

The detection and quantification of dopamine was performed in a similar protocol as previously described [26]. Prior to dialysate analysis, a standard curve consisted of a series of known dopamine concentrations was established for every experiment. The dopamine assay was performed on UHPLC system (Thermo Scientific™ Dionex™ UltiMate™ 3000) equipped with ECD-3000RS (Electrochemical cell: 6011RS, Thermo Scientific). Samples were injected automatically and separated on an analytical column Acclaim RSLC PA2, 250 × 2.1 mm, 2.2 μm (Thermo Scientific, 074814). For dopamine assay, the applied potential for analytical cell was set as + 220 mV. The UHPLC system was operated at 400 μL/min for 10 mins at 30 °C using test mobile phase (Thermo Scientific). The column temperature was set to 30 °C. The concentration of dopamine was measured relative to standard solutions using Chromeleon 7.2 Chromatography Data System (Dionex, Thermo Scientific).

### Locomotor apparatus

Open-field chambers (Med Associates Inc., St. Albans, VT, USA) were used to measure the locomotor activity of the experimental subjects. The dimensions of each open-field chamber were 43 cm long × 43 cm wide × 30 cm high. The walls of each open-field chamber were made of Plexiglas with a ventilated top-cover. There were six 16-beam infrared arrays mounted along the walls each chamber, allowing automated measurements of horizontal locomotor activity (Program Activity Monitor version 5.08; Med Associates Inc.) A custom-built system of 16 clear polycarbonate boxes (45 × 20 × 25 cm^3^) was also used in this study. The length of each box had an array of 11 externally mounted infrared photodetectors spaced 4 cm apart and 2 cm above the cage floor. Photobeam interruptions were recorded as ambulatory counts on the computer system.

### Co-immunoprecipitation and Western blot

Co-immunoprecipitation and Western Blot analyses were performed as previously described [27]. Briefly, rat striatal tissues were homogenized in ice-cold lysis buffer (50 mM Tris, 150 mM NaCl, 2 mM EDTA, 1% NP-40, 0.5% sodium deoxycholate, pH = 7.4, with protease inhibitor (Sigma-Aldrich)), and rotated for 1 h at 4 °C. After being centrifuged at 12,000 g for 10 min, the total solubilized protein extract was yielded in the supernatant. 1000 μg solubilized protein extracted from rat striatal tissue was incubated in the presence of anti-D2R antibody (Proteintech Group, rabbit, catalogue# 55084–1-AP) or control IgG (Merck Millipore, rabbit, catalogue# 12–370), together with protein A/G plus agarose (Santa Cruz Biotechnology) overnight at 4 °C. Pellets were washed, boiled for 5 min in SDS sample buffer (Bio-Rad) + 2-Mercaptoethanol (a reducing agent; Sigma-Aldrich) and subjected to SDS-PAGE. 50~ 100 μg of protein extracted from tissue was used as a control in each experiment. After transfer of proteins onto nitrocellulose, membranes were Western blotted with the primary antibodies specified below. The intensity of protein level was quantified by densitometry (software: Image Lab, Bio-Rad). The antibodies used include anti-D2R (Santa Cruz Biotechnology, mouse, catalogue# sc-5303) and anti-DAT (Santa Cruz Biotechnology, rat, catalogue# sc-32258). More information, the D2R antibody from Santa Cruz Biotechnology was previously validated using D2 KO animals and the data have been published [27]. The other D2R antibody from Proteintech was validated by another research group using cell culture and siRNA against Drd2 [28].

### Data analysis

Unless stated otherwise, behavioral data and co-immunoprecipitation data were analyzed by two-way ANOVA with/without repeated measures using IBM SPSS Statistics 21 (IBM Corporation). Co-immunoprecipitation data was analyzed by t-test, or one-way ANOVA followed by Bonferroni post-hoc test. All graphs presented in this paper were constructed by GraphPad Prism 5. All data were presented as Mean ± SEM unless otherwise stated.

## Results

### The disruption of D2R-DAT stimulates locomotor activity

As aforementioned, we developed an interfering peptide (TAT-DAT_NT_) to disrupt the interaction between DAT and D2R, which can increase locomotor activity in mice [25]. Given the therapeutic effects of DAT blockers and stimulant agents (e.g. methylphenidate) in ADHD, we hypothesize that the disruption of D2R-DAT protein complex might be a new treatment means for ADHD by enhancing the dopaminergic neurotransmission. Thus, the ultimate goal of the current study was to investigate the potential effects of TAT-DAT_NT_ in an animal model of ADHD. In the current literature, the spontaneously hypertensive rats (SHR) is one of the most widely-used and validated animal models of ADHD [29, 30]. Before we investigated the effects of TAT-DAT_NT_ in SHR rats, we hoped to rule out the possibility that TAT-DAT_NT_ may act differently in rats compare to mice. Therefore, we began our study by confirming whether the disruption of D2R-DAT protein complex by TAT-DAT_NT_ enhances the locomotor activity in rats.

**Fig. 1:**
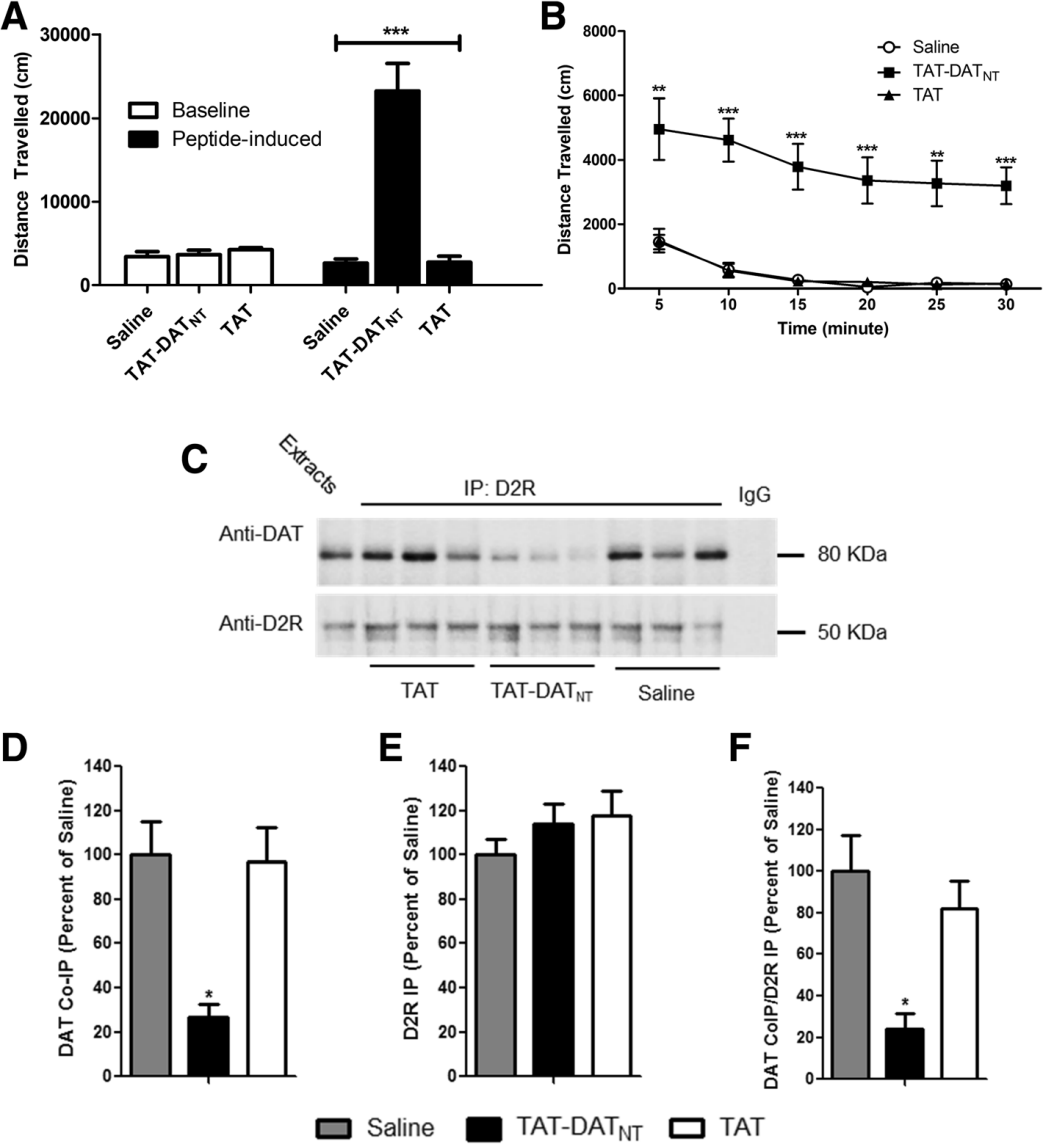
The disruption of D2R-DAT protein complex stimulates voluntary movement of SD rats. **a**-**b** The effects of TAT-DAT_NT_ peptide treatment on locomotor behavior (*n* = 6–8 animals per group). **a** Thirty minutes after the peptide administration, SD rats treated with TAT-DAT_NT_ (at 40 nmol, *i.c.v.*) exhibited a significantly higher level of locomotor activity compared to those treated with saline or TAT (*p* < 0.001). **b** The stimulant effects of TAT-DAT_NT_ were significant across all six time intervals compared to both saline and TAT, indicating that the difference in locomotor activity is unlikely novelty-driven. **c-f** The effects of TAT-DAT_NT_ on the D2R-DAT protein interaction (*n* = 3 per group). **c** Co-immunoprecipitation showed that TAT-DAT_NT_ disrupts the D2R-DAT complex in SD rats, as compared to those from TAT- or saline-injected group. **d-f** Densitometric analysis of DAT co-immunoprecipitation (DAT CoIP) and D2R immunoprecipitation (D2R IP) from striatal lysate of SD rats injected with saline, TAT, or TAT-DAT_NT_ peptide. Results for each sample are presented as the ratio of the saline group. Data were analyzed by one-way ANOVA followed by Tukey’s test. *p < 0.05, **p < 0.01, ***p < 0.001. Data are shown as mean ± S.E.M

In Fig. 1a, normal SD rats were placed in open field boxes to compare baseline and peptide-induced locomotor activities among the three treatment groups (*n* = 6–8 animals per group). The three groups did not differ in baseline level (*p* = NS). Subsequently, we administered their respective treatments (i.e. saline, TAT-DAT_NT_ or TAT) intracranially. Thirty minutes after the administration, we tracked their locomotor activities for another 30 min and discovered that SD rats treated with TAT-DAT_NT_ (40 nmol) exhibited a higher level of locomotion activity. Two-way ANOVA analysis revealed significant treatment effect on locomotor activity (F_2, 44_ = 29.0, *p* < 0.001), and Bonferroni post-hoc analysis confirmed that TAT-DAT_NT_ treatment were statistically different from both saline and TAT treatment (*p* < 0.001).

The locomotor data were further divided and analyzed at each of the six five-minute intervals as shown in Fig. 1b. Two-way repeated measure ANOVA analysis revealed a significant time (F_1.39, 24.863_ = 8.02, *p* < 0.01) and treatment (F_2, 19_ = 26.827, *p* < 0.001) effect on locomotor activity. However, no significant time × treatment interaction (F_10, 96_ = 0.291, *p* = 0.891) effect were detected. Furthermore, Bonferroni post-hoc analysis also confirmed that the TAT-DAT_NT_ peptide significantly elevated voluntary movement across all six time-points compared to both saline and TAT control peptide (*p* < 0.01 or less; Fig. 1b).

To confirm that the stimulant effect of TAT-DAT_NT_ was due to its disruptive actions on D2R-DAT protein complexes, we performed co-immunoprecipitation using striatal brain tissues from SD rats that received the treatments. Ideally, we would like to use tissues from nucleus accumbens core (NAc), a region responsible for locomotor behavior, for this co-immunoprecipitation experiment, but NAc alone might not yield enough proteins to practically perform this assay. Since NAc is also considered a part of striatum, we decided to use striatal tissues, which include NAc, for our co-immunoprecipitation experiment. Co-immunoprecipitation showed that TAT-DAT_NT_ disrupted the D2R-DAT complex in SD rats, as compared to those from TAT- or saline-injected groups (Fig. 1c). In this co-immunoprecipitation assay, we used total protein extracts as a positive control and naïve IgG as a negative control. The direct immuno-precipitated D2R (i.e. the lower panel of Fig. 1c) serves as a control to ensure that equal amount of D2R was present in each sample and that the differences in co-immunoprecipitation of DAT were due to changes in the degree of D2R-DAT interaction, rather than antibody artifacts. In Fig. 1d, densitometric analysis revealed that the co-immunoprecipitation of DAT (DAT CoIP) by D2R antibody was significantly lower in SD rats injection with TAT-DAT_NT_ as compared to those injected with saline and TAT alone (one-way ANOVA with Bonferroni post-hoc analysis, F_2,8_ = 10.398, *p* < 0.05). We also examined the level of immunoprecipitation of D2R (D2R IP) and found no difference among the three groups (one-way ANOVA, F_2, 8_ = 3.87, *p* = NS; Fig. 1e). After determining the ratio between DAT CoIP and D2R IP for each sample, we further confirmed the reduction of DAT CoIP by TAT-DAT_NT_ was not due to any potential difference in D2R IP (one-way ANOVA with Bonferroni post-hoc analysis, F_2, 8_ = 9.01, *p* < 0.05; Fig. 1f). These results showed that TAT-DAT_NT_, by disrupting the D2R-DAT interaction, leads to increased locomotor behavior in SD rats.

### AMPT-mediated dopamine depletion model

Our next objective was to investigate whether TAT-DAT_NT_ can continue to promote locomotor activity even when endogenous dopamine level was low. Before we could continue our investigation, however, we wished to validate a published dopamine depletion model [25]. This model was adapted from McDougall et al. [25], they reported that animals pre-treated with AMPT showed lower locomotor activity, which was further confirmed by another research group [31]. We chose this partial depletion model because hypo-dopaminergic activity, rather than complete lack of dopamine, is implicated with the patho-physiology of ADHD. Therefore, we believed this AMPT-mediated partial dopamine depletion model was more suitable for our study rather than a complete deletion model, before we moved to a more widely-used model of ADHD --- spontaneously hypertensive rats.

**Fig. 2:**
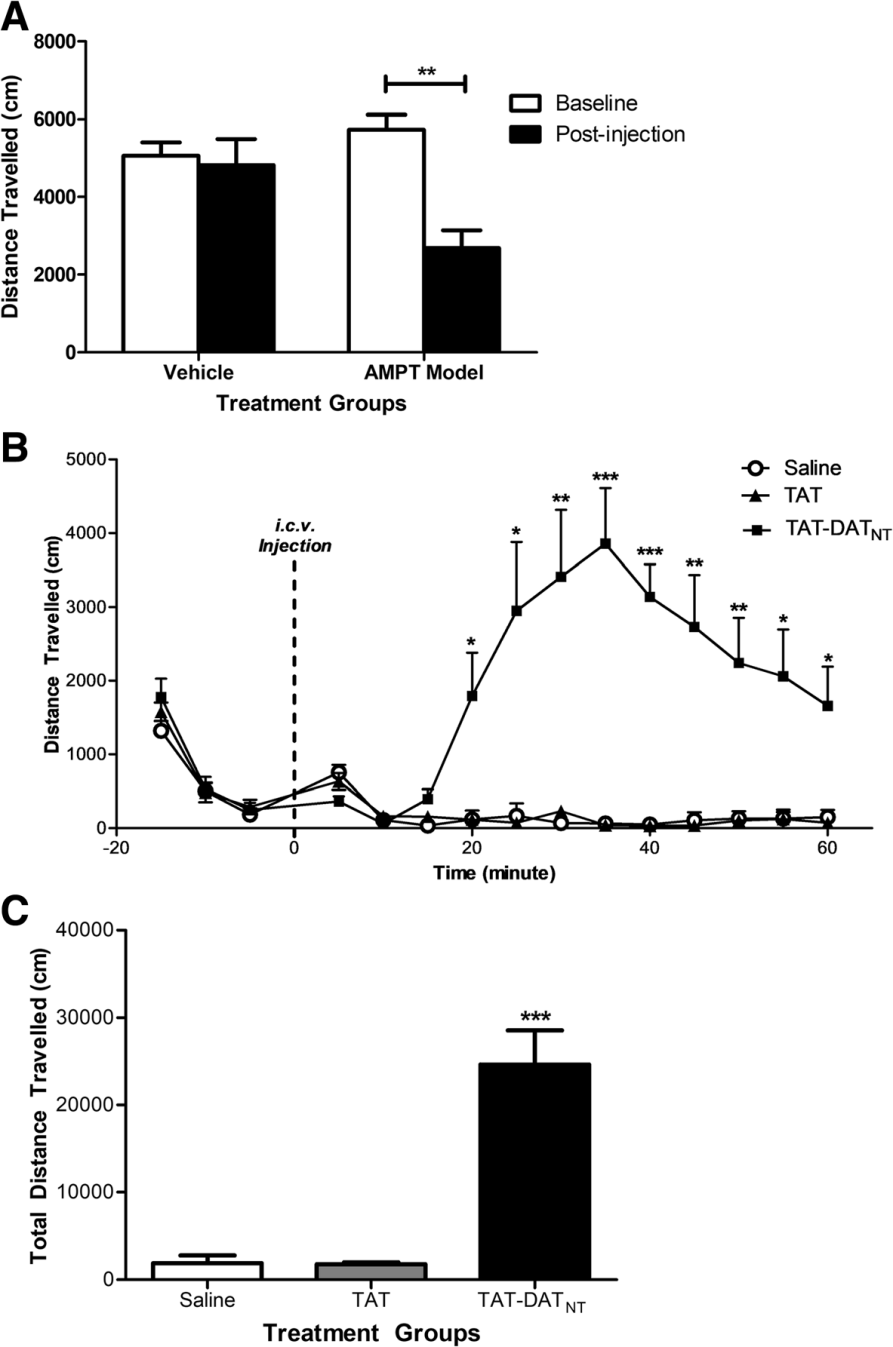
The disruption of D2R-DAT by TAT-DAT_NT_ rescues the locomotor impairment imposed by AMPT-mediated dopamine depletion. **a** The AMPT treatment significantly impaired locomotor activity compared to vehicle (*n* = 5–6 per group). **b** A timewise graph illustrates the locomotor activities of dopamine-depleted animals injected with saline, TAT or TAT-DAT_NT_ (*n* = 6–8 per group). **c** TAT-DAT_NT_ (40 nmol, *i.c.v.*) significantly elevated the locomotor movement in the dopamine-depleted animals. All data were presented as mean ± SEM. *, *p* < 0.05; **, *p* < 0.01; ***, *p* < 0.001

In order to validate this dopamine-depletion model, we had animals from both vehicle and AMPT groups placed in open-field chambers on three consecutive days to record their baseline locomotor activity. Following the injection of either vehicle or AMPT, animals were returned to open field boxes to track their locomotor activities for 30 min. Two-way ANOVA reported significant injection effect (F_1, 18_ = 11.02, *p* < 0.01). The vehicle group did not differ in locomotor activity between baseline and post-injection (Bonferroni post-hoc analysis, *p* = NS; Fig. 2a), whereas the post-injection locomotor activity in the AMPT group was significantly lower than its baseline level, approximately a 50% reduction (Fig. 2a; *p* < 0.01).

### The effects of D2R-DAT disruption in DA-depleted animals

We next examined whether dopamine depletion can affect the ability of TAT-DAT_NT_ to promote locomotor behavior. Animals (*n* = 6–8 per group) were treated with AMPT to cause dopamine depletion and were placed in open-field chambers for 15 min. Then, animals received their *i.c.v.* injections (i.e. saline, TAT, TAT-DAT_NT_) and were immediately returned to the open-field chambers for 1 hour.

One-way ANOVA analysis revealed significant treatment effect on the total locomotor activity (F_2, 20_ = 26.10, *p* < 0.001; Fig. 2c), where animals treated with TAT-DAT_NT_ exhibited a significantly higher level of locomotion (Bonferroni post-hoc analysis, *p* < 0.001).

The hourly locomotor activity was further analyzed at each five-minute interval (Fig. 2b). Two-way ANOVA with repeated measures confirmed treatment effect (F_2, 18_ = 29.63, *p* < 0.001) and time effect (F_2.66, 47.85_ = 4.58, *p* < 0.01). TAT-DAT_NT_ began to elevate locomotor behavior approximately 20 min after administration and its stimulant effect lasted throughout the remainder of the experiment (Bonferroni post-hoc analysis, *p* < 0.05 or less). Consequently, this experiment confirmed that dopamine depletion does not abolish the ability of D2R-DAT disruption to promote dopamine-driven behavior.

### The effects of D2R-DAT disruption on extracellular dopamine

**Fig. 3:**
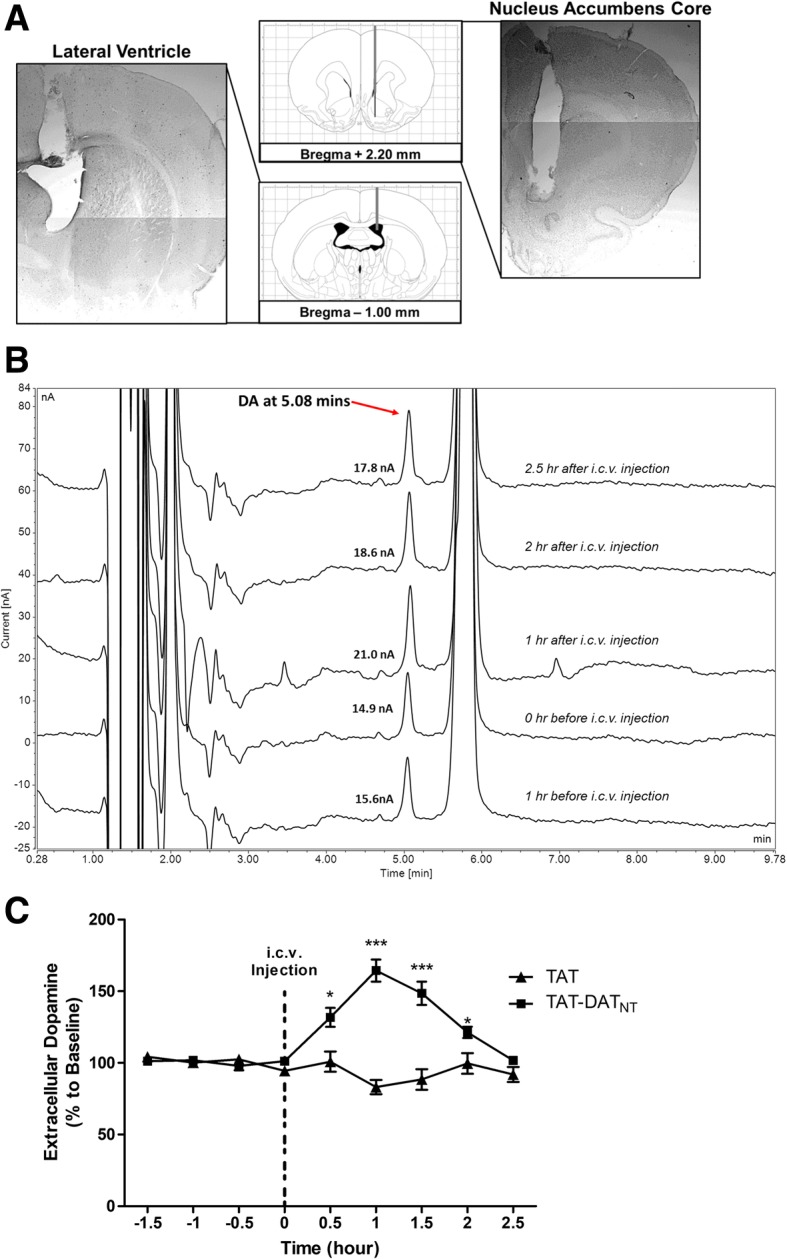
The stimulant effect of D2R-DAT disruption is due to the rise of extracellular dopamine level in SD rats. **a** Schematic illustration and cresyl violet staining confirmation of cannula placement into both the lateral ventricle and nucleus accumbens core. The grey bars represent the cannula, whereas the black bar represents the 2-mm probe membrane. b Time-course raw traces of high performance liquid chromatography before and after the administration of TAT-DAT_NT_ was illustrated. The retention time of dopamine was determined to be at 5.08 min. c TAT-DAT_NT_ (40 nmol, *i.c.v.*) started to increase the extracellular dopamine level half an hour after its administration and such effect remained significant approximately for 2 hours (*n* = 5–6). The dotted line represented the time of intra-cranial injection. All data were presented as percent dopamine to baseline (%) ± SEM. *p < 0.05, **p < 0.01 and ***p < 0.001

Next, we performed an in vivo microdialysis study to investigate whether the disruption of D2R-DAT protein complex by TAT-DAT_NT_ increases extracellular level the nucleus accumbens core (NAc), part of the ventral striatum, which can explain the stimulant effects of TAT-DAT_NT_ observed previously. It has been widely reported that nucleus accumbens core (NAc) is essential for mediating locomotor movements in rodents [32–34]. As shown in Fig. 3a, we placed a guide cannula into the lateral ventricle for the administration of either TAT or TAT-DAT_NT_ peptide, whereas a microdialysis cannula was implanted into the nucleus accumbens core for the collection of dialysates. We used high-performance liquid chromatography to analyze the dopamine content in each dialysate, and the retention time of dopamine was determined to be at 5.08 min (Fig. 3b). Prior to the injection of either peptide treatment, dialysates were continually collected for 2 hours to determine the basal extracellular dopamine level. As illustrated in Fig. 3c, both groups (*n* = 5–6 per group) displayed a similar baseline of extracellular dopamine level (*p* = NS). Two-way ANOVA with repeated measures revealed that no significant treatment (F_1, 9_ = 0.112, *p* = NS), no time effect (F_3, 27_ = 1.174, *p* = NS) and no treatment × time effect (F_3, 27_ = 1.926, *p* = NS).

Following the *i.c.v.* administration, TAT-DAT_NT_, at the dose of 40 nmol (*i.c.v.*), increased the extracellular NAc dopamine level (Fig. 3b). Two-way ANOVA with repeated measures reported significant time effect (F_4, 36_ = 9.23, *p* < 0.001), treatment effect (F_1, 9_ = 38.31, *p* < 0.001) and time × treatment effect (F_4, 36_ = 18.65, *p* < 0.001). Compared to the treatment of TAT, the *i.c.v.* injection of TAT-DAT_NT_ significantly elevated extracellular NAc dopamine content and the significant increase lasted for 2 hours (Bonferroni post-hoc analysis, *p* < 0.05 or less; Fig. 3c). Overall, this in vivo microdialysis study provided strong evidence that the disruption of D2R-DAT by the TAT-DAT_NT_ peptide results in an increased extracellular dopamine level.

### The Effects of TAT-DAT_NT_ on Hyperactivity in SHR rats

The spontaneously hypertensive rat (SHR), a commonly used animal model of attention deficit/hyperactivity disorder (ADHD) [35], is genetically bred from progenitor Wistar Kyoto rat (WKY) [36]. The SHR rats exhibit similar behavioral characteristics as children with ADHD such as hyperactivity [29, 37, 38]. Since the current stimulant treatments (e.g. methylphenidate) for ADHD all strengthen synaptic dopamine signaling [39], we examined the effects of the TAT-DAT_NT_ on the hyperactivity of SHR rats and we hypothesized that this peptide will reverse such hyperactivity by disrupting the D2R-DAT interaction.

**Fig. 4:**
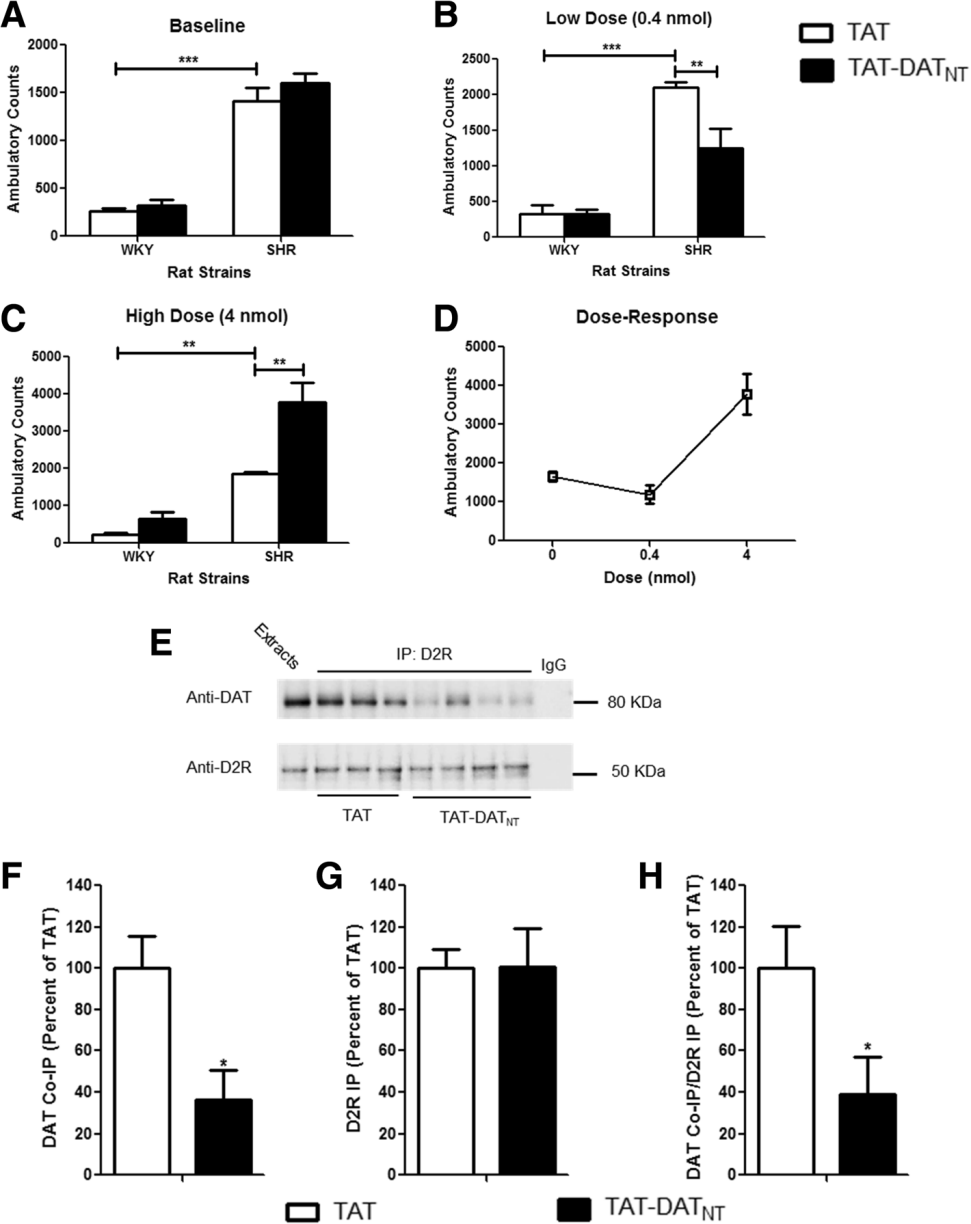
TAT-DATNT alleviated the hyperactivity of spontaneously hypertension rats. **a-d**, The effects of TAT-DAT_NT_ peptide treatment on locomotor behavior in WKY and SHR rats (n = 6–8 per group). **a** SHR rats manifested a significantly higher level of baseline locomotor activity compared to the control strain WKY rats (*p* < 0.001). **b** TAT-DAT_NT_, at 0.4 nmol, alleviated the hyperactivity of SHR rats as compared to the TAT control (*p* < 0.01). **c** TAT-DAT_NT_ at 4 nmol exacerbated the hyperactivity of SHR rats compared to the TAT-treated SHR rats (*p* < 0.01). **d** TAT-DAT_NT_ displayed an U-shaped dose-response curve in SHR rats. **e-h** The effects of TAT-DAT_NT_ (at 0.4 nmol) on the D2R-DAT interaction in SHR rats (n = 3 for TAT, and 4 for TAT-DAT_NT_). **e** TAT-DAT_NT_ reduced the level of D2R-DAT complex in SHR rats, as compared to those from TAT-injected group. **f-h** Densitometric analysis of DAT co-immunoprecipitation (CoIP) and D2R immunoprecipitation (D2R IP) from striatal lysate of SHR rats injected with TAT, or TAT-DAT_NT_ peptide. Results for each sample are presented as the ratio of the TAT samples. Data were analyzed by t-test. *p < 0.05, **p < 0.01, ***p < 0.001. Data are shown as mean ± S.E.M

WKY rats were included in this study as a negative control strain for the hyperactivity of SHR rats. All WKY and SHR rats were placed in the locomotor boxes for two consecutive days to determine their baseline locomotor activities. As illustrated in Fig. 4a, SHR rats exhibited locomotor hyperactivity compared to WKY rats, which two-way ANOVA confirmed significant strain effect (F_1, 20_ = 233.2, *p* < 0.001).

To avoid hypertension as a potential confounding factor, we decided to test the SHR rats and WKY rats at 4 weeks old, and therefore they were a lot smaller compared to the SD rats in the previous experiment. As a result, we chose a lower dose (0.4 nmol, *i.c.v.*) of TAT-DAT_NT_ to test its effects on the locomotor behavior in these animals. In Fig. 4b, TAT-DAT_NT_ significantly decreased the locomotor activity in SHR compared to TAT (*p* < 0.01) but had no effects in WKY rats (*p* = NS). At 4 nmol, however, TAT-DAT_NT_ exacerbated the hyperactivity of SHR rats (*p* < 0.01; Fig. 4c). Altogether, TAT-DAT_NT_ displayed a U-shaped dose-response curve in SHR rats as shown in Fig. 4d, where it alleviated hyperactivity at low dose, but worsened such behavioral abnormality at a higher dose.

To verify the peptide effects were due to the disruption of D2R-DAT complex, we performed co-immunoprecipitation using striatal brain tissues from SHR rats that received the treatments. As illustrated in Fig. 4e, the administration of TAT-DAT_NT_ reduced the level of D2R-DAT interaction in SHR rats, as compared to those treated with TAT. The densitometric analysis quantified that the TAT-DAT_NT_ peptide significantly reduced the level of D2R-DAT complex as compared to TAT group (t_5_ = 3.69, *p* < 0.05; Fig. 4f). We also examined the level of immunoprecipitation of D2R (D2R IP) and found no difference between the two groups (t_5_ = 0.36, *p* = NS; Fig. 4g). Finally, we also determined the ratio between DAT CoIP and D2R IP, and confirmed that the lower DAT CoIP in animals treated with TAT-DAT_NT_ was not due to any potential difference in D2R IP (t_5_ = 2.79, *p* < 0.05; Fig. 4h). These data confirmed that the TAT-DAT_NT_ peptide can rescue the locomotor hyperactivity in SHR rats by disrupting the D2R-DAT interaction.

### The effects of TAT-DAT_NT_ on spontaneous alternation behavior (SAB) in SHR rats

Aside from hyperactivity, SHR rats also display lower spontaneous alternation behavior (SAB) in a Y-maze test [40–42]. We were interested in examining whether the administration of TAT-DAT_NT_ can improve such behavior deficits in SHR rats using the same Y-maze test. In this test, animals could explore all three arms freely (Additional file 1: Figure S1A). A spontaneous alternation is made when an animal visit three different arms in three consecutive arm entries.

SHR rats treated with TAT-DAT_NT_ (0.4 nmol, *i.c.v.*) showed a significantly higher percentage of spontaneous alternation behavior compared to their TAT counterparts (two-tailed t-test, t_10_ = 2.422, *p* < 0.05; Additional file 1: Figure S1B). As illustrated in Additional file 1: Figure S1C, animals from both TAT and TAT-DAT_NT_ did not differ in time spent in each duration (two-way ANOVA with repeated measures, F_1, 10_ = 0.321, *p* = NS), suggesting that the differences in spontaneous alternation behavior were unlikely due to confounding factors such as anxiety.

## Discussion

Dopamine transporter (DAT) facilitates the reuptake of dopamine from the synaptic cleft into the presynaptic neuronal terminal, playing a crucial role in regulating dopaminergic signaling. The importance of DAT in dopaminergic signaling was further reiterated by Jones et al. as they suggested that the lifespan of synaptic dopamine prolongs by approximately 300 times and the synaptic dopamine content increases by 5 times in the absence of DAT [43]. The actions of DAT can be regulated by a wide range of factors and cellular events and can be summarized in three main categories: 1) ligand-binding (e.g. substrates and inhibitors), 2) enzymatic modification, and 3) protein-protein interaction.

On the same presynaptic terminus of dopaminergic neurons, there are dopamine D2 receptors (D2R) serving as a presynaptic auto-inhibitory receptor, which is implicated in the regulation of DAT functions. We previously discovered that, regardless of its activation state, D2R can enhance the membrane expression of DAT and consequently DAT-mediated reuptake of dopamine through their direct protein-protein interaction [23]. Structurally, this interaction is mediated through the first 15 amino acids of the N-terminus of DAT, which sequence was used to generate the interfering peptide (i.e. TAT-DAT_NT_) in this study. Interestingly, the first 22 amino acids of the N-terminus are essential for protein kinase C-mediated phosphorylation of DAT [44]. Therefore, it is possible that D2R promotes the actions of DAT by masking the N-terminus from the regulatory phosphorylation. Although our previous study of D2R-DAT interaction was conducted in mouse tissues, we have confirmed the existence of D2R-DAT interaction using rat striatal tissue and that TAT-DAT_NT_ peptide was able to disrupt the D2R-DAT interaction in this study (Fig. 1c). We also observed that TAT-DAT_NT_ decrease dopamine reuptake in rat primary cultures in our previous study [23]. Moreover, the region of DAT (M_1_ - V_15_: MSKSKCSVGPMSSVV) responsible for mediating the interaction with D2R is identical between mice and rats, which the same amino acid sequence was used to synthesized TAT-DAT_NT_. Thus, the ability of TAT-DAT_NT_ to disrupt the D2R-DAT interaction should not be altered, which was confirmed by our results in Fig. 1c. In addition, using the BLAST tool of PubMed (N.I.H.), we confirmed that this amino acid sequence of TAT-DAT_NT_ was only found in dopamine transporter, but not in norepinephrine transporter (NET) and serotonin transporter (SERT) (species: *Rattus norvegicus*). Therefore, we do not expect that TAT-DAT_NT_ will affect NET and SERT.

Since dopamine is critically involved in movement initiation and facilitation through its actions on both direct and indirect pathways in the basal ganglia circuitry [45–47], it is plausible to presume that a higher extracellular dopamine level should equate behavioral alterations such as an elevated level of voluntary movements. This is exactly the effects TAT-DAT_NT_ observed in this study. The disruption of D2R-DAT by the intracranial administration of TAT-DAT_NT_ results in a significantly higher level of locomotor activity than the control groups (*p* < 0.001; Fig. 1a). The locomotor activities of animals from the TAT-DAT_NT_ group were significantly elevated at all six time-points compared to both saline and TAT groups (*p* < 0.001; Fig. 1b). It prompted that the differences in locomotor behavior were not due to novelty since the novelty-induced hyperactivity is usually observed within the first 5–10 min and dramatically normalize afterwards. Moreover, all the animals habituated to the open-field boxes on three consecutive days prior to the testing, therefore minimizing the effects of novelty as a potential confounding factor. Such dramatic enhancement in locomotor behavior further emphasized the critical roles of DAT-mediated reuptake in the termination of dopaminergic neurotransmission. Similarly, hyperactivity due to elevated dopamine content in the synapse has been reported in mice lacking DAT [48] and rats with pharmacological blockade of DAT [49, 50].

We were also interested in whether the D2R-DAT disruption can strengthen dopamine signaling even under hypo-dopaminergic signaling. Hence, we examined the effects of TAT-DAT_NT_ in an acute dopamine depletion model. AMPT exerts its inhibitory actions on tyrosine hydroxylase, which will lead to a severe impairment in the endogenous production of dopamine [25, 31]. In this model, the disruption of D2R-DAT also sufficed to alleviate the AMPT-induced impairment on locomotor behavior (*p* < 0.001; Fig. 2b and c).

Based on the data from both normal and dopamine-depleted animals, we hoped to confirm that the disruption of D2R-DAT stimulates locomotor behavior by directly enhancing dopaminergic neurotransmission. Therefore, we decided to employ in vivo microdialysis to ensure the behavior effects observed in rodents were due to a rise in extracellular dopamine level. We targeted the nucleus accumbens core (NAc) because this region is considered to essentially mediate voluntary locomotor movement in rodents [32–34]. As we hypothesized, the interference by TAT-DAT_NT,_ at the dose of 40 nmol, rendered a significant rise in extracellular dopamine (*p* < 0.001), and which lasted for approximately 2 hours (Fig. 3c). Since we do not expect that TAT-DAT_NT_ would have opposite effects at different doses, we did not perform in vivo microdialysis for different doses of TAT-DAT_NT_.

As mentioned in the introduction, dysregulation of dopaminergic system has been implicated in attention deficit hyperactivity disorder (ADHD), which is characterized by hyperactivity, impaired working memory, impulsivity and inattention [51–53]. It is postulated that ADHD may result from deficits in the dopaminergic system in cortical brain structures such as the prefrontal cortex, subcortical areas such as striatum [54, 55]. Particularly, the dopamine transporter (DAT) has drawn a lot of attention as a suitable candidate for treating ADHD. For instance, the mainstay medication for ADHD is stimulants that block DAT to achieve their therapeutic effects [56–58]. In addition, neuroimaging studies of patients with ADHD reported an elevated level of striatal dopamine transporter activity, which is reverted by the administration of methylphenidate [59, 60]. Given the implication of dopamine dysregulation and dopamine transporter in ADHD, we are curious about whether the D2R-DAT protein complex will prove to be a viable therapeutic target for ADHD. Therefore, we investigated whether the disruption of D2R-DAT can exert any beneficial effects on the ADHD-like symptoms (i.e. hyperactivity and impaired working memory) in a widely used rodent model of ADHD --- spontaneously hypertensive rats (SHR) [35, 36]. It is widely believed that methylphenidate produces its therapeutic effects in ADHD by increasing dopamine level, which is similar to the actions of TAT-DAT_NT_ as supported the microdialysis data (Fig. 3C). TAT-DAT_NT_ alleviated hyperactivity in SHR rats (Fig. 4B), suggesting that enhancement in the dopaminergic neurotransmission can exert therapeutic effects on symptoms of ADHD. However, there are reports that, unlike its low dose, high dose of methylphenidate fails to offer the same therapeutic effects on hyperactivity in SHR rats [41, 61]. We also made similar observations at a higher dose of TAT-DAT_NT_ (i.e. 4.0 nmol) in SHR rats, demonstrating a U-shaped dose-response curve on the hyperactivity in SHR (Fig. 4D). We believe this U-shaped dose-response curve occurs due to the possibility that low-dosed TAT-DAT_NT_ returns the abnormally low level of dopaminergic signaling to the physiological range, and therefore normalizes the hyperactivity of SHR rats. However, when there is too much TAT-DAT_NT_, it may excessively enhance dopaminergic neurotransmission and rather exerts its stimulant effects as observed in SHR rats (Fig. 4c).

WKY rats were included in the study as a control strain for SHR rats. Although one study reported a higher level of DAT in the striatum of SHR rats than WKY rats when they were 2-week old [62], overall no significant difference in the levels of D2R or DAT was reported between SHR and WKY rats. TAT-DAT_NT_, at neither 0.4 nmol nor 4.0 nmol, elicited any effects on locomotor activity in the WKY rats. Similar to the effects of TAT-DAT_NT_, the effects of methylphenidate on WKY rats are also dose-dependent [63, 64]. At a dose (i.e. 0.6 mg/kg) that is effective in SHR rats, methylphenidate dose not trigger any changes in the locomotor activity of WKY rats [63]. Methylphenidate can stimulate locomotor activity in WKY rats only when a much higher dose (e.g 2.5–10 mg/kg) is given. The doses of TAT-DAT_NT_ tested in WKY rats were 0.4 nmol and 4.0 nmol per animals in our study, which therefore may not suffice to trigger stimulant effects in WKY rats.

Aside from hyperactivity, SHR rats are readily reported to exhibit low level of spontaneous alternation behavior in the Y-maze test [41, 42]. Here, we reported that TAT-DAT_NT_ significantly enhanced spontaneous alternation behavior in SHR rats compared to those received TAT (*p* < 0.05; Additonal file 1: Figure S1B). Such improvement may be due to enhanced dopaminergic neurotransmission as studies have suggested that dopaminergic neurotransmission is involved in the manifestation of spontaneous alternation behavior. The chronic administration of haloperidol (i.e. a dopaminergic antagonist) decreases spontaneous alternation behavior [65], whereas methylphenidate (i.e. a dopaminergic stimulant) increases the same behavior [66].

It is worth noting that we used TAT peptide as the control peptide in this study rather than the second N-terminus fragment of DAT (A_16_ – P_26_) from our previous study [23]. It has been reported that the N-terminus of DAT is essential for protein kinase C-mediated phosphorylation on serine residues [67], and the first 22 amino acids from the N-terminus of DAT eliminate phosphorylation of DAT in response to PKC activation [44]. Since the second fragment of DAT covers part of the first 22 amino acids and contains serine residue, we were concerned that it may have unknown effects on DAT. Therefore, we chose to use TAT peptide as the control peptide instead. TAT peptide is also commonly used as a control peptide by other research groups [68–70] as well as our research group [71, 72]. On the other hand, there is a possibility that TAT-DAT_NT,_ which main action is to disrupt the D2R-DAT interaction, may affect phosphorylation of DAT and other DAT-associated protein interactions. The investigation of TAT-DAT_NT_’s effects on DAT phosphorylation and other DAT-associated protein complexes will be the focus of our future studies.

Current therapies for ADHD block DAT and completely inhibit the reuptake of dopamine. Although they yield great clinical outcomes, they are also associated with serious adverse effects due to their complete blockade of DAT [39]. Here, we reported that a protein-protein interaction between dopamine D2 receptor (D2R) and dopamine transporter (DAT) provides a new way to modulate the activities of DAT and dopamine reuptake. Unlike conventional DAT blockers such as methylphenidate, the disruption of D2R-DAT leads to decreased membrane expression of DAT, while leaves the remaining DAT functional to facilitate the reuptake of dopamine [23]. We found that the disruption of this protein complex by an interfering peptide, namely TAT-DAT_NT_, increases extracellular dopamine level and therefore stimulate locomotor behavior in rats. Furthermore, at an appropriate dose, TAT-DAT_NT_ alleviates hyperactivity and improves working memory in spontaneously hypertensive rats, an animal model of ADHD. Unlike typical DAT blockers, TAT-DAT_NT_ specifically targets the D2R-DAT interaction and should possess no affinity for other transporters [39]. Although there may be concerns with its bioavailability in the brain, we have previously proved that intra-nasal delivery can be a viable route to counter this potential obstacle [73]. Hopefully, our findings altogether expand our current understanding of DAT regulation and its implication in ADHD and add values to future therapeutic development.

## Additional files

Additional file 1: **Figure S1**. Low-dosed TAT-DAT_NT_ improved attention deficits in SHR rats. **A**, Schematic illustration of the Y-maze used in the study. **B,** TAT-DAT_NT_ (0.4 nmol, *i.c.v.*) promoted spontaneously alternation behavior in SHR rats compared to the TAT control peptide (n = 6 for each group). **C,** Neither TAT nor TAT-DAT_NT_ caused SHR rats to spend more time or stay away from any arms (n = 6 per group). All data were presented as mean ± SEM. *p < 0.05 (TIF 2213 kb).

## Abbreviations

ADHD: Attention Deficit/Hyperactivity Disorder
D2R: Dopamine D2 Receptor
DAT: Dopamine Transporter
SD: Sprague-Dawley Rats
SHR: Spontaneously Hypertensive Rats
WKY: Wistar Kyoto rats
NAc: Nucleus accumbens core
