# Supplementary figures and images for: Development of a peptide targeting dopamine transporter to improve ADHD-like deficits
Source: Mol Brain. 2018 Nov 9;11:66. doi: 10.1186/s13041-018-0409-0 (PMC6234781; doi:10.1186/s13041-018-0409-0)

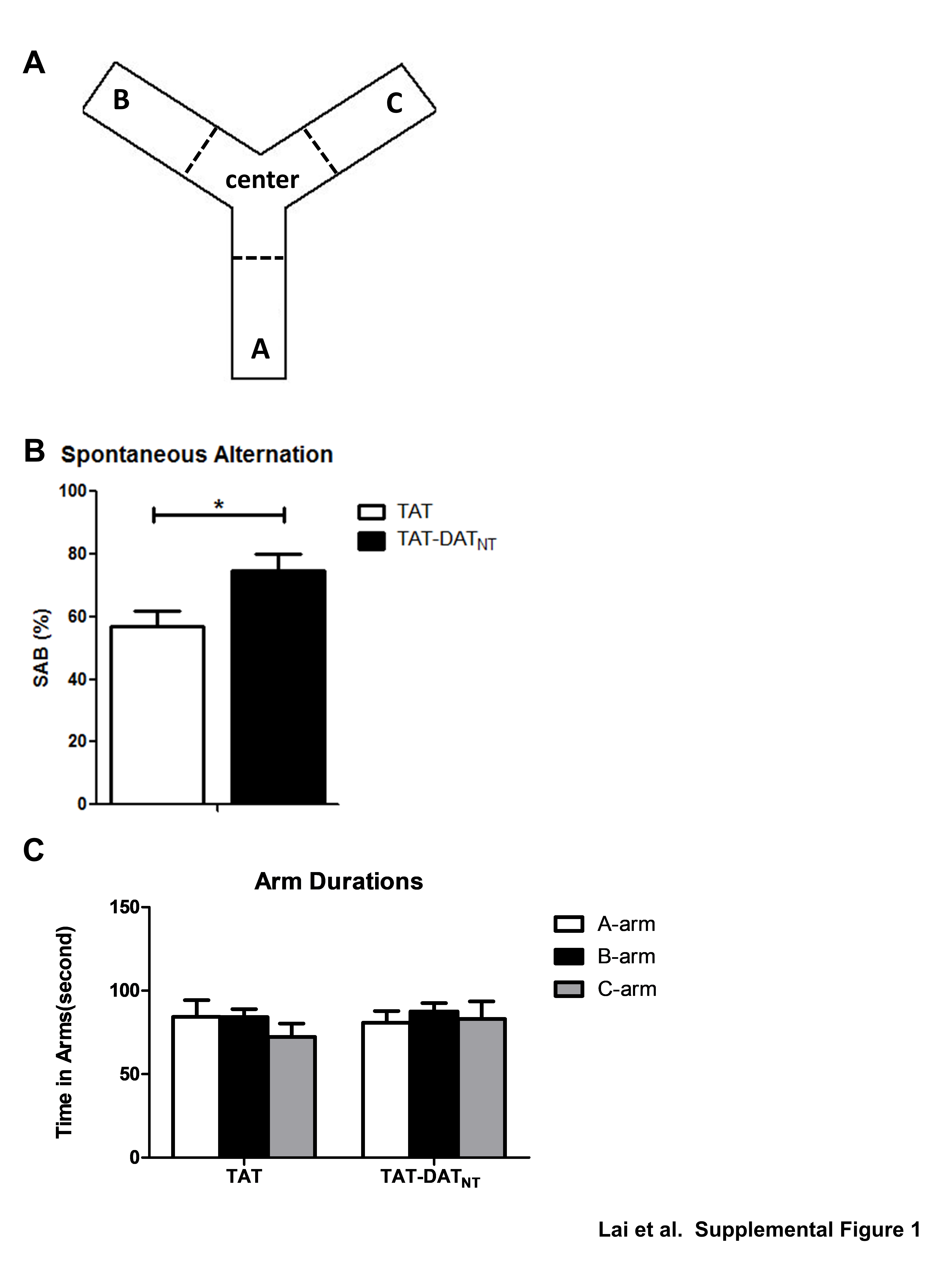

Supplement: Supplementary file 1 — Figure S1. Low-dosed TAT-DATNT improved attention deficits in SHR rats. A, Schematic illustration of the Y-maze used in the study. B, TAT-DATNT (0.4 nmol, i.c.v.) promoted spontaneously alternation behavior in SHR rats compared to the TAT control peptide (n = 6 for each group). C, Neither TAT nor TAT-DATNT caused SHR rats to spend more time or stay away from any arms (n = 6 per group). All data were presented as mean ± SEM. *p < 0.05 (TIF 2213 kb). [file 13041_2018_409_MOESM1_ESM.tif]
